# 4-Dimethyl­amino-*N*′-(2-meth­oxy­benzyl­idene)benzohydrazide

**DOI:** 10.1107/S1600536811025220

**Published:** 2011-07-02

**Authors:** Fu Su, Zheng-Gui Gu, Jun Lin

**Affiliations:** a Jiangsu Centre of Extraction Seperation Engineering Technology, College of Chemistry and Materials Science, Nanjing Normal University, Nanjing 210046, People’s Republic of China

## Abstract

In the title mol­ecule, C_17_H_19_N_3_O_2_, the dihedral angle between the two benzene rings is 14.05 (15)°. In the crystal, mol­ecules are linked through inter­molecular N—H⋯O hydrogen bonds, forming chains along *b*.

## Related literature

For the biological properties of hydrazones, see: Ajani *et al.* (2010 ▶); Zhang *et al.* (2010 ▶); Angelusiu *et al.* (2010 ▶). For similar structures, see: Huang & Wu (2010 ▶); Khaledi *et al.* (2010 ▶); Zhou & Yang (2010 ▶); Ji & Lu (2010 ▶); Singh & Singh (2010 ▶); Ahmad *et al.* (2010 ▶); Su *et al.* (2011 ▶).
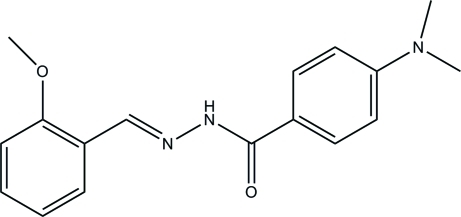


## Experimental

### 

#### Crystal data

C_17_H_19_N_3_O_2_
*M* *_r_* = 297.35
Orthorhombic, 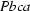

*a* = 16.065 (3) Å
*b* = 7.946 (2) Å
*c* = 24.534 (3) Å
*V* = 3131.8 (11) Å^3^
*Z* = 8
Mo *K*α radiation
μ = 0.09 mm^−1^
*T* = 298 K
0.13 × 0.10 × 0.08 mm

#### Data collection

Bruker SMART CCD area-detector diffractometer
Absorption correction: multi-scan (*SADABS*; Bruker, 2001 ▶) *T*_min_ = 0.989, *T*_max_ = 0.993
13733 measured reflections
2800 independent reflections
1570 reflections with *I* > 2σ(*I*)
*R*_int_ = 0.094

#### Refinement

*R*[*F* ^2^ > 2σ(*F* ^2^)] = 0.064
*wR*(*F* ^2^) = 0.140
*S* = 0.99
2800 reflections
203 parameters
H-atom parameters constrained
Δρ_max_ = 0.20 e Å^−3^
Δρ_min_ = −0.18 e Å^−3^

### 

Data collection: *SMART* (Bruker, 2007 ▶); cell refinement: *SAINT* (Bruker, 2007 ▶); data reduction: *SAINT*; program(s) used to solve structure: *SHELXS97* (Sheldrick, 2008 ▶); program(s) used to refine structure: *SHELXL97* (Sheldrick, 2008 ▶); molecular graphics: *SHELXTL* (Sheldrick, 2008 ▶); software used to prepare material for publication: *SHELXTL*.

## Supplementary Material

Crystal structure: contains datablock(s) global, I. DOI: 10.1107/S1600536811025220/su2286sup1.cif

Structure factors: contains datablock(s) I. DOI: 10.1107/S1600536811025220/su2286Isup2.hkl

Supplementary material file. DOI: 10.1107/S1600536811025220/su2286Isup3.cml

Additional supplementary materials:  crystallographic information; 3D view; checkCIF report

## Figures and Tables

**Table 1:** Hydrogen-bond geometry (Å, °)

*D*—H⋯*A*	*D*—H	H⋯*A*	*D*⋯*A*	*D*—H⋯*A*
N2—H2⋯O2^i^	0.91	2.07	2.966 (3)	169

Symmetry code: (i) 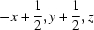.

**Fig. 1.**
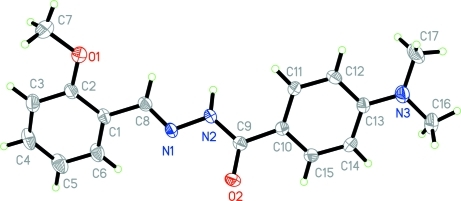
The molecular structure of the title molecule showing 30% probability displacement ellipsoids and the atomic numbering. Hydrogen bonds are drawn as dashed lines.

**Fig. 2.**
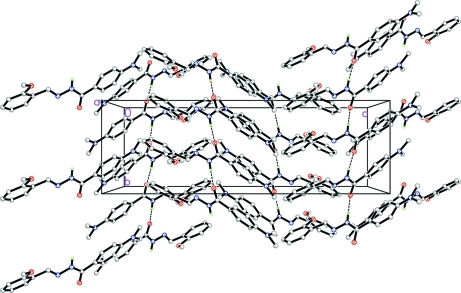
Crystal packing of the title compound, viewed down the a axis. Intermolecular interactions are drawn as dashed lines.

## supplementary crystallographic information

### Comment

In 2010, much attention has been focused on the biological properties of hydrazone compounds (Ajani *et al.*, 2010; Zhang *et al.*, 2010; Angelusiu *et al.*, 2010). The crystal structures of a number of hydrazone compounds have also been determined (Huang & Wu, 2010; Khaledi *et al.*, 2010; Zhou & Yang, 2010; Ji & Lu, 2010; Singh & Singh, 2010; Ahmad *et al.*, 2010). Herein, we report on the synthesis and crystal structure of a new hydrazone compound, prepared by the reaction of 2-methoxybenzaldehyde with 4-dimethylaminobenzohydrazide.

In the molecule of the title compound, Fig. 1, the dihedral angle between the two benzene rings is 14.05 (15) °. All the bond values are comparable with those in the similar compound, *N'*-(4-Diethylamino-2-hydroxybenzylidene)-4-(dimethylamino)benzohydrazide methanol monosolvate, we reported recently (Su *et al.*, 2011).

In the crystal structure, the hydrazone molecules are linked through intermolecular N–H···O hydrogen bonds (Table 1), to form 1D chains along *b* (Fig. 2).

### Experimental

The reaction of 2-methoxybenzaldehyde (0.136 g, 1 mmol) with 4-dimethylaminobenzohydrazide (0.179 g, 1 mmol) in 30 ml methanol at room temperature afforded the title compound. Colourless single crystals were formed by gradual evaporation of the solution in air.

### Refinement

The amino H atom was located in a difference Fourier map and refined with the N–H distance restrained to be 0.90 (1) Å. The remaining H atoms were positioned geometrically (C–H = 0.93-0.96 Å) and refined as riding with *U*_iso_(H) = 1.2*U*_eq_(C) or 1.5*U*_eq_(C_methyl_).

### Crystal data

**Table:**

C_17_H_19_N_3_O_2_	*F*(000) = 1264
*M**_r_* = 297.35	*D*_x_ = 1.261 Mg m^−^^3^
Orthorhombic, *P**b**c**a*	Mo *K*α radiation, λ = 0.71073 Å
Hall symbol: -P 2ac 2ab	Cell parameters from 1584 reflections
*a* = 16.065 (3) Å	θ = 2.5–24.5°
*b* = 7.946 (2) Å	µ = 0.09 mm^−^^1^
*c* = 24.534 (3) Å	*T* = 298 K
*V* = 3131.8 (11) Å^3^	Block, colourless
*Z* = 8	0.13 × 0.10 × 0.08 mm

### Data collection

**Table:**

Bruker SMART CCD area-detector diffractometer	2800 independent reflections
Radiation source: fine-focus sealed tube	1570 reflections with *I* > 2σ(*I*)
graphite	*R*_int_ = 0.094
ω scans	θ_max_ = 25.1°, θ_min_ = 2.1°
Absorption correction: multi-scan (*SADABS*; Bruker, 2001)	*h* = −19→14
*T*_min_ = 0.989, *T*_max_ = 0.993	*k* = −9→9
13733 measured reflections	*l* = −29→28

### Refinement

**Table:**

Refinement on *F*^2^	Secondary atom site location: difference Fourier map
Least-squares matrix: full	Hydrogen site location: inferred from neighbouring sites
*R*[*F*^2^ > 2σ(*F*^2^)] = 0.064	H-atom parameters constrained
*wR*(*F*^2^) = 0.140	*w* = 1/[σ^2^(*F*_o_^2^) + (0.0579*P*)^2^] where *P* = (*F*_o_^2^ + 2*F*_c_^2^)/3
*S* = 0.99	(Δ/σ)_max_ < 0.001
2800 reflections	Δρ_max_ = 0.20 e Å^−^^3^
203 parameters	Δρ_min_ = −0.18 e Å^−^^3^
0 restraints	Extinction correction: *SHELXL97* (Sheldrick, 2008), Fc^*^=kFc[1+0.001xFc^2^λ^3^/sin(2θ)]^-1/4^
Primary atom site location: structure-invariant direct methods	Extinction coefficient: 0.0028 (7)

### Special details

**Table:**

Geometry. All esds (except the esd in the dihedral angle between two l.s. planes) are estimated using the full covariance matrix. The cell esds are taken into account individually in the estimation of esds in distances, angles and torsion angles; correlations between esds in cell parameters are only used when they are defined by crystal symmetry. An approximate (isotropic) treatment of cell esds is used for estimating esds involving l.s. planes.
Refinement. Refinement of F^2^ against ALL reflections. The weighted R-factor wR and goodness of fit S are based on F^2^, conventional R-factors R are based on F, with F set to zero for negative F^2^. The threshold expression of F^2^ > 2sigma(F^2^) is used only for calculating R-factors(gt) etc. and is not relevant to the choice of reflections for refinement. R-factors based on F^2^ are statistically about twice as large as those based on F, and R- factors based on ALL data will be even larger.

### Fractional atomic coordinates and isotropic or equivalent isotropic displacement parameters (Å^2^)

**Table:**

	*x*	*y*	*z*	*U*_iso_*/*U*_eq_	
O1	0.48383 (13)	0.6587 (3)	0.24567 (9)	0.0662 (7)	
O2	0.14952 (11)	0.4522 (2)	0.11421 (8)	0.0472 (6)	
N1	0.26607 (14)	0.5707 (3)	0.17978 (9)	0.0416 (6)	
N2	0.24365 (13)	0.6531 (3)	0.13259 (8)	0.0416 (6)	
H2	0.2698	0.7527	0.1273	0.050*	
N3	0.05256 (15)	0.9526 (3)	−0.07705 (10)	0.0528 (7)	
C1	0.34787 (17)	0.5653 (4)	0.25970 (11)	0.0411 (7)	
C2	0.42874 (18)	0.5812 (4)	0.27952 (11)	0.0436 (7)	
C3	0.4498 (2)	0.5152 (4)	0.32985 (13)	0.0559 (9)	
H3	0.5038	0.5257	0.3431	0.067*	
C4	0.3908 (2)	0.4350 (5)	0.35968 (13)	0.0665 (10)	
H4	0.4047	0.3937	0.3940	0.080*	
C5	0.3123 (2)	0.4135 (5)	0.34093 (14)	0.0695 (11)	
H5	0.2736	0.3542	0.3615	0.083*	
C6	0.29024 (19)	0.4800 (4)	0.29113 (13)	0.0588 (9)	
H6	0.2360	0.4674	0.2785	0.071*	
C7	0.5688 (2)	0.6547 (5)	0.26050 (16)	0.0847 (12)	
H7A	0.5767	0.7171	0.2936	0.127*	
H7B	0.6016	0.7041	0.2320	0.127*	
H7C	0.5858	0.5401	0.2660	0.127*	
C8	0.32433 (17)	0.6371 (3)	0.20687 (11)	0.0411 (7)	
H8	0.3521	0.7310	0.1934	0.049*	
C9	0.18162 (16)	0.5881 (4)	0.10239 (11)	0.0364 (7)	
C10	0.15273 (16)	0.6857 (3)	0.05514 (10)	0.0353 (7)	
C11	0.20007 (16)	0.8036 (4)	0.02693 (10)	0.0410 (8)	
H11	0.2546	0.8242	0.0378	0.049*	
C12	0.16804 (18)	0.8897 (4)	−0.01651 (11)	0.0460 (8)	
H12	0.2016	0.9663	−0.0349	0.055*	
C13	0.08536 (17)	0.8651 (4)	−0.03401 (11)	0.0387 (7)	
C14	0.03883 (17)	0.7460 (4)	−0.00569 (11)	0.0428 (8)	
H14	−0.0160	0.7254	−0.0160	0.051*	
C15	0.07247 (17)	0.6587 (3)	0.03703 (11)	0.0405 (7)	
H15	0.0401	0.5781	0.0545	0.049*	
C16	−0.03085 (19)	0.9181 (4)	−0.09651 (12)	0.0630 (10)	
H16A	−0.0354	0.8013	−0.1060	0.095*	
H16B	−0.0421	0.9861	−0.1280	0.095*	
H16C	−0.0703	0.9442	−0.0684	0.095*	
C17	0.1000 (2)	1.0754 (5)	−0.10564 (15)	0.0850 (12)	
H17A	0.1213	1.1568	−0.0803	0.128*	
H17B	0.0653	1.1308	−0.1319	0.128*	
H17C	0.1456	1.0215	−0.1240	0.128*	

### Atomic displacement parameters (Å^2^)

**Table:**

	*U*^11^	*U*^22^	*U*^33^	*U*^12^	*U*^13^	*U*^23^
O1	0.0498 (14)	0.0811 (17)	0.0678 (14)	−0.0141 (13)	−0.0137 (12)	0.0283 (13)
O2	0.0488 (12)	0.0363 (12)	0.0566 (13)	−0.0065 (10)	−0.0030 (10)	0.0106 (10)
N1	0.0426 (14)	0.0413 (15)	0.0410 (14)	0.0018 (13)	−0.0046 (12)	0.0138 (12)
N2	0.0451 (14)	0.0384 (15)	0.0413 (13)	−0.0043 (12)	−0.0076 (12)	0.0132 (11)
N3	0.0532 (16)	0.0579 (18)	0.0473 (15)	0.0045 (14)	−0.0077 (13)	0.0092 (14)
C1	0.0442 (18)	0.0409 (18)	0.0383 (16)	0.0080 (15)	0.0006 (14)	0.0076 (14)
C2	0.0500 (19)	0.0403 (18)	0.0404 (17)	0.0013 (15)	−0.0037 (15)	0.0065 (15)
C3	0.064 (2)	0.053 (2)	0.050 (2)	0.0052 (18)	−0.0167 (17)	0.0033 (17)
C4	0.082 (3)	0.077 (3)	0.0406 (19)	0.023 (2)	−0.0012 (19)	0.0167 (19)
C5	0.059 (2)	0.089 (3)	0.061 (2)	0.017 (2)	0.0184 (19)	0.033 (2)
C6	0.0423 (19)	0.075 (2)	0.059 (2)	0.0147 (17)	0.0046 (16)	0.0217 (19)
C7	0.052 (2)	0.089 (3)	0.113 (3)	−0.012 (2)	−0.017 (2)	0.027 (2)
C8	0.0428 (17)	0.0364 (17)	0.0442 (17)	0.0034 (15)	0.0008 (14)	0.0058 (15)
C9	0.0333 (16)	0.0327 (17)	0.0432 (17)	0.0046 (14)	0.0030 (13)	0.0020 (14)
C10	0.0389 (17)	0.0317 (16)	0.0352 (15)	0.0019 (13)	−0.0008 (13)	−0.0013 (13)
C11	0.0367 (16)	0.0471 (19)	0.0391 (17)	−0.0054 (14)	−0.0056 (14)	0.0044 (14)
C12	0.0526 (19)	0.0443 (19)	0.0412 (17)	−0.0085 (15)	0.0032 (15)	0.0094 (15)
C13	0.0444 (17)	0.0364 (18)	0.0353 (15)	0.0056 (15)	−0.0035 (14)	−0.0031 (14)
C14	0.0404 (16)	0.0459 (19)	0.0423 (17)	−0.0048 (15)	−0.0070 (14)	−0.0044 (15)
C15	0.0424 (18)	0.0375 (18)	0.0414 (17)	−0.0033 (14)	−0.0007 (14)	0.0001 (14)
C16	0.069 (2)	0.068 (2)	0.0512 (19)	0.0169 (19)	−0.0200 (17)	−0.0021 (18)
C17	0.077 (3)	0.099 (3)	0.079 (3)	−0.002 (2)	−0.007 (2)	0.050 (2)

### Geometric parameters (Å, °)

**Table:**

O1—C2	1.361 (3)	C7—H7A	0.9600
O1—C7	1.413 (3)	C7—H7B	0.9600
O2—C9	1.231 (3)	C7—H7C	0.9600
N1—C8	1.263 (3)	C8—H8	0.9300
N1—N2	1.378 (3)	C9—C10	1.470 (4)
N2—C9	1.345 (3)	C10—C15	1.381 (3)
N2—H2	0.9056	C10—C11	1.391 (3)
N3—C13	1.369 (3)	C11—C12	1.367 (4)
N3—C17	1.424 (4)	C11—H11	0.9300
N3—C16	1.449 (4)	C12—C13	1.410 (4)
C1—C6	1.383 (4)	C12—H12	0.9300
C1—C2	1.393 (4)	C13—C14	1.392 (4)
C1—C8	1.466 (4)	C14—C15	1.368 (4)
C2—C3	1.383 (4)	C14—H14	0.9300
C3—C4	1.357 (4)	C15—H15	0.9300
C3—H3	0.9300	C16—H16A	0.9600
C4—C5	1.353 (4)	C16—H16B	0.9600
C4—H4	0.9300	C16—H16C	0.9600
C5—C6	1.378 (4)	C17—H17A	0.9600
C5—H5	0.9300	C17—H17B	0.9600
C6—H6	0.9300	C17—H17C	0.9600
			
C2—O1—C7	117.4 (2)	C1—C8—H8	120.2
C8—N1—N2	115.9 (2)	O2—C9—N2	121.1 (2)
C9—N2—N1	118.3 (2)	O2—C9—C10	121.1 (3)
C9—N2—H2	127.0	N2—C9—C10	117.8 (2)
N1—N2—H2	114.4	C15—C10—C11	117.1 (2)
C13—N3—C17	121.4 (3)	C15—C10—C9	117.8 (2)
C13—N3—C16	121.0 (3)	C11—C10—C9	125.1 (2)
C17—N3—C16	117.6 (3)	C12—C11—C10	121.3 (3)
C6—C1—C2	118.3 (3)	C12—C11—H11	119.4
C6—C1—C8	120.7 (3)	C10—C11—H11	119.4
C2—C1—C8	120.9 (3)	C11—C12—C13	121.5 (3)
O1—C2—C3	123.8 (3)	C11—C12—H12	119.2
O1—C2—C1	115.7 (2)	C13—C12—H12	119.2
C3—C2—C1	120.4 (3)	N3—C13—C14	121.6 (3)
C4—C3—C2	119.2 (3)	N3—C13—C12	121.8 (3)
C4—C3—H3	120.4	C14—C13—C12	116.6 (2)
C2—C3—H3	120.4	C15—C14—C13	121.0 (3)
C5—C4—C3	121.8 (3)	C15—C14—H14	119.5
C5—C4—H4	119.1	C13—C14—H14	119.5
C3—C4—H4	119.1	C14—C15—C10	122.5 (3)
C4—C5—C6	119.5 (3)	C14—C15—H15	118.8
C4—C5—H5	120.2	C10—C15—H15	118.8
C6—C5—H5	120.2	N3—C16—H16A	109.5
C5—C6—C1	120.7 (3)	N3—C16—H16B	109.5
C5—C6—H6	119.7	H16A—C16—H16B	109.5
C1—C6—H6	119.7	N3—C16—H16C	109.5
O1—C7—H7A	109.5	H16A—C16—H16C	109.5
O1—C7—H7B	109.5	H16B—C16—H16C	109.5
H7A—C7—H7B	109.5	N3—C17—H17A	109.5
O1—C7—H7C	109.5	N3—C17—H17B	109.5
H7A—C7—H7C	109.5	H17A—C17—H17B	109.5
H7B—C7—H7C	109.5	N3—C17—H17C	109.5
N1—C8—C1	119.6 (3)	H17A—C17—H17C	109.5
N1—C8—H8	120.2	H17B—C17—H17C	109.5
			
C8—N1—N2—C9	−179.6 (2)	O2—C9—C10—C15	23.9 (4)
C7—O1—C2—C3	7.1 (4)	N2—C9—C10—C15	−155.4 (2)
C7—O1—C2—C1	−170.6 (3)	O2—C9—C10—C11	−156.0 (3)
C6—C1—C2—O1	176.7 (3)	N2—C9—C10—C11	24.7 (4)
C8—C1—C2—O1	−3.2 (4)	C15—C10—C11—C12	0.6 (4)
C6—C1—C2—C3	−1.1 (4)	C9—C10—C11—C12	−179.6 (3)
C8—C1—C2—C3	179.0 (3)	C10—C11—C12—C13	1.1 (4)
O1—C2—C3—C4	−177.7 (3)	C17—N3—C13—C14	179.4 (3)
C1—C2—C3—C4	0.0 (5)	C16—N3—C13—C14	−3.1 (4)
C2—C3—C4—C5	1.9 (5)	C17—N3—C13—C12	−1.2 (4)
C3—C4—C5—C6	−2.6 (6)	C16—N3—C13—C12	176.2 (3)
C4—C5—C6—C1	1.4 (5)	C11—C12—C13—N3	179.1 (3)
C2—C1—C6—C5	0.4 (5)	C11—C12—C13—C14	−1.5 (4)
C8—C1—C6—C5	−179.6 (3)	N3—C13—C14—C15	179.6 (3)
N2—N1—C8—C1	175.3 (2)	C12—C13—C14—C15	0.2 (4)
C6—C1—C8—N1	−28.0 (4)	C13—C14—C15—C10	1.5 (4)
C2—C1—C8—N1	152.0 (3)	C11—C10—C15—C14	−1.8 (4)
N1—N2—C9—O2	−5.2 (4)	C9—C10—C15—C14	178.3 (2)
N1—N2—C9—C10	174.1 (2)		

### Hydrogen-bond geometry (Å, °)

**Table:**

*D*—H···*A*	*D*—H	H···*A*	*D*···*A*	*D*—H···*A*
N2—H2···O2^i^	0.91	2.07	2.966 (3)	169

Symmetry codes: (i) −*x*+1/2, *y*+1/2, *z*.
